# Supplementary material for: Are carbon emissions trading and green financial instruments synergistic? -Comprehensive quantitative research based on content analysis
Source: PLoS One. 2024 Mar 7;19(3):e0298601. doi: 10.1371/journal.pone.0298601 (PMC10919601; doi:10.1371/journal.pone.0298601)
Supplement: S1 Data — (ZIP) [file pone.0298601.s001.zip › Data/地级市Z_F工作报告.zip!/║┌┴·╜¡/╥┴┤║╩╨/└┤╫╘╣1⁄2╓┌║┼í╛┬φ┐╦╩2╛▌═°í┐_2009.docx]

各位代表：

现在，我代表市人民政府，向大会报告工作，请予审议，并请政协委员和列席会议的同志提出意见。

一、关于2008年的工作

2008年，是我市发展进程中极不平凡的一年。在市委的正确领导下，市政府坚持以科学发展观为指导，积极践行服务人民、造福人民、让改革发展成果更多更广地惠及人民的诺言，团结和依靠全市人民，大力实施“生态立市、产业兴市”战略，一以贯之保生态，励精图治谋发展，竭尽全力保民生，圆满完成了市十二届人大二次会议确定的各项任务。

(一）经济发展好中加快，速度质量同步提升。全市生产总值实现179亿元，为计划的106%，同比增长12.4%，已连续五年保持两位数增长。全口径财政总收入突破10亿元大关，达到 11.1亿元，为预算的159.2%，同比增长37.9%，其中地方财政收入完成5.96亿元，为预算的157.7%，同比增长31.7%。财政总支出完成39.3亿元，为预算的116.3%，同比增长29.8%。全社会固定资产投资完成69.3亿元，为计划的111%，同比增长38.2％。31个投资5000万元以上的省市级重点项目相继开工，争取到国家扩大内需的政策性投资21项1.74亿元。全市规模以上工业实现增加值30亿元，为计划的101%，同比增长12%。社会消费品零售总额实现40.4亿元，为计划的107%，同比增长21.3%。循环经济增加值实现15.2亿元，同比增长31.3%。万元GDP综合能耗下降4.5%。全年各项预期目标均超额完成，全市经济开始步入加快发展的新阶段。

（二）资源保护深入开展，生态建设取得新成效。小兴安岭生态功能区被纳入省八大经济区之一，我市的生态建设在全省发展中的地位显著提升。修订完善了《小兴安岭生态功能区建设规划》，开展了红松保护国际交流与合作活动，在禁伐天然红松的基础上，又将椴树、色树、三大硬阔等珍贵濒危树种纳入禁伐序列，对林蛙、蓝莓果等野生动植物实行了重点保护，使生态保护重点由单一树种向多树种、由林上向林下、由植物向动物扩大延伸，生态保护体系进一步完善。全市建成不同级别和类型的自然保护区20处、62万公顷，其中新青被国家林业局批准为“国家湿地公园试点”单位，白头鹤自然保护区被评为省级保护区。基地化保护野生蓝莓果6000公顷。汤旺河公园被批准为我国首个建设试点的国家公园。深入落实“严管林”措施，资源林政案件进一步减少。建立实施了木材最低销售价制度，有效地避免了木材资源效益流失。依法收回未按规定造林的宜林“两荒”和被蚕食的林地3479.9公顷，累计完成退耕还林、还湿、还草31万亩，更新造林5.56万亩，荒山荒地造林6.2万亩，全民义务植树357万株，防治林业有害生物面积312万亩。完成土地整理复垦项目11项，1990公顷坑洼地恢复了耕种功能。积极主动地开展了停止森林主伐的测算与申报工作。高度重视森防工作，出台并严格落实《进一步加强森防工作的决定》，加大投入，完善制度，提高了综合防控能力，杜绝了重特大森林火灾的发生。

(三）接替产业日益壮大，经济转型步伐加快。以生态旅游为主的第三产业、绿色能源、特色种养与加工、冶金建材及矿产开发、木材精深加工等接替产业不断壮大，增加值实现126.2亿元，同比增长13.9%，占GDP的比重达70.5%，成为我市经济发展的主导力量。金融业健康发展，贷款余额77亿元，同比增长4.8%，支持了地方经济发展。森林生态旅游业发展势头强劲，接待游客337万人次，收入实现15.1亿元，同比增长25.7%，我市被评为“中国2008年度最具开发价值的旅游资源城市”。农业又获丰收，粮食总产量达到64万吨，为计划的106.8%，同比增长10.2%。非国有经济实现增加值131.2亿元，同比增长12.5%，已成为我市最具有活力的经济增长点。

（四）改革开放不断深化，发展活力与日俱增。发端于我市的国有林区林权制度改革试点被评为“全国改革开放30年创新案例”，写入了中央1号文件，继续扩大林改试点被写入了十七届三中全会《决定》，林改这一重大举措得到了中央的充分肯定。晨明水泥厂、带岭林业造纸厂政策性破产依法办理终结。浩良河水泥公司股权成功转让西钢，资产重组后的浩水公司实现了当月盈利。光明集团公司连续多年的产权纠纷难题得到依法解决，避免了下市风险，有望重振雄风。对外开放取得新成效。对俄合作不断升级，全年在俄采伐木材92万立方米，同比增长16%，对俄劳务输出5000人。招商引资到位资金实现51.2亿元，同比增长40%；实际利用外资1497万美元，同比增长27.3%；外贸进出口完成2.2亿美元，同比增长63.4%，三项指标均创历史最好水平。

(五）城乡建设力度加大，人居环境明显改善。城市基础设施建设完成投资3.56亿元，同比增加1.06亿元。新建改造城市道路67.6万平方米，开发建设住宅楼房87万平方米，完成漏雨楼房“平改坡”7.6万平方米，修缮了300余栋老旧楼房损毁严重的共用门窗，新增城镇供水管线7220延长米，新增集中供热31.9万平方米，新增绿地90.3公顷。严格土地管理，开展了土地市场清理整顿，城镇土地市场日趋规范。大力实施棚户区改造工程，改造面积完成54.8万平方米。林都机场主体工程正在紧锣密鼓地建设，伊绥高速公路伊春段也已破土动工，几代林区人梦想的立体交通网络即将变为现实。兴安湖水库顺利截流，纳入松花江流域水污染防治的中心城区和南岔污水处理厂等6个项目全部开工建设。空气质量达标率达98.9%。伊春的天更蓝、水更清、城市更美了。小城镇建设力度加大，城镇面貌大为改观。新农村建设取得成效，建设农村公路748.6公里，铺装村屯、林场所硬化道路292.1公里，改造泥草房2159户，新建砖瓦房10万平方米，建设户用沼气池3000个，有线电视入户率达52%以上，农村和林场所的生产生活条件明显改善。

（六）社会事业全面进步，民生保障得到加强。科技创新步伐加快，取得技术成果27项，高新技术产业产值同比增长48.8%。办学水平明显提高，撤并农村、林场所学校21所，投资5422万元新建维修改建校舍14.7万平方米，高考平均分在全省排名由三年前的倒数第二升至第七位。就医条件得到改善，三级医疗服务机构全面建立，社区卫生服务人口覆盖率达到80%以上，新农合参合率达95%。开展了“兴安健康行”活动，为30余万人进行了免费体检。文化事业繁荣发展，创作出《林都伊春》等一大批优秀文艺精品，“大森林之声”系列文化活动丰富多彩，有线电视数字化开始运营。全民健身活动广泛开展，竞技体育在国内外赛事中屡创佳绩。稳控物价取得成效，居民消费价格低于全省1个百分点。人口计划生育、妇女儿童、民族宗教、外事侨务、防震减灾、人防、双拥等工作也取得了较好成绩。坚持把就业作为民生之本，实现城镇就业再就业5.2万人，新增就业4.4万人，开发公益性岗位1393个，登记失业率4.0 %，低于省控指标0.6 个百分点。职工自营经济从业人员不断增加，已经成为职工群众就业增收的一条重要渠道。社会保障覆盖面不断扩大，各类社会保险金100%发放，14.2万困难群众享受到了最低生活保障。争取和安排资金2.4亿元提高了养老保险和最低保障标准，失业保险金和抚恤补助发放标准也进一步提高，2.9万户低保家庭得到了廉租住房保障。补发了森工企业职工历史拖欠的3.8亿元工资，解决了2.1万人的饮水安全问题。第四轮“扶贫解困”工作取得阶段性成果，脱贫率达75%。地方财政对民生的投入大幅增加，占财政总支出比例达到27.5%，同比增长8.3%。城镇居民人均可支配收入达到8724元，同比增长8.9%；农民人均纯收入达到5371元，同比增长14.9%。城乡居民人均储蓄存款达1.4万元，同比增长17%，增速为近10年来最快的一年。

(七）文明程度逐步提高，和谐局面更加巩固。“四城联创”取得突破性进展，我市创建全国文明城市工作被中央文明委命名为先进城市，创建国家卫生城市获取申报资格。思想道德建设成效显著，被评为全省唯一的全国未成年人思想道德建设先进城市。民主法制建设不断加强，坚持向人大及其常委会报告工作和向政协通报情况制度，自觉接受法律监督和民主监督，建立了提案、议案办理制度，办理人大议案、建议、批评和意见55件，政协提案95件，做到了件件有着落、事事有回音。加强了与民主党派、工商联和人民团体的联系。依法治市力度不断加大，“平安伊春”建设成效显著，严重刑事案件同比下降18%，安全生产完成了省下达的事故控制指标，食品药品安全得到保障，信访总量同比下降14%，社会大局持续稳定。

各位代表、同志们，过去的一年，我市在发展形势不断变化、国际金融危机突如其来等不利因素诸多的情况下，能取得这些成绩，实属不易。这是省委、省政府和市委正确领导的结果，是市人大和市政协有效监督、大力支持的结果，是离退休老同志和社会各界切实关心、密切配合的结果，是全市上下凝心聚力、真抓实干的结果。在此，我代表市人民政府，向辛勤工作在各条战线的全市各族人民和关心支持伊春建设的各位领导、朋友们致以崇高的敬意和衷心的感谢！

回顾一年的工作，我们也清醒地认识到，与科学发展观的总要求比较，与其它走在我们前面的地区对照，与人民群众的期盼衡量，我们在工作中，仍然存在一些亟待解决的矛盾和问题。主要是：生态历史欠账多，资源恢复周期长，破坏生态资源的现象仍时有发生；接续替代产业发展水平不高，抗击市场风险的能力弱；发展环境不尽人意，服务不够到位，诚信还有缺失，融资渠道窄，信贷投入少；在工资水平、教育、医疗、供热、供水等方面与人民群众日益增长的需求还有很大差距，限于政策及财力等因素，部分群众的困难问题还没有从根本上得到解决；人才缺乏的问题突出，特别是重点行业、重点领域的人才严重不足；个别部门职能转变滞后，部分政府工作人员思想解放不够，改革意识、责任意识、公仆意识不强，创新力、执行力较差，工作效率不高，铺张浪费、大手大脚现象不同程度地存在。有些问题，群众的意见较大。

各位代表、同志们，过去的成绩，是做好今年工作的基础，我们将倍加珍惜；对存在的问题，我们一定高度重视，认真加以解决，也希望大家更加有力地监督、支持，推动政府的工作。

二、关于2009年的工作任务

2009年，将是我市既面临严峻挑战又充满难得机遇的一年。目前，国际金融危机对我市发展的影响仍未缓解，出口市场萎缩、国内市场低迷影响到了大豆、玉米、木耳、家具等我市传统优势农林产品销售，多年销售顺畅的木材也出现了滞销现象。多数企业效益下滑，对经济增长和人民群众的生产生活带来了很大影响，也加大了财政收支矛盾。何时能够摆脱国际金融危机的负面影响，现在还无法预期。客观上的不利因素和发展中累积的矛盾相互交织，今年很可能是我市进入市场经济时代以来最为困难的一年，对此，我们必须有足够的思想准备和充分的应对之策。另一方面，对面临的有利因素，我们更要紧紧把握，增强战胜困难的信心和勇气。综合分析，我们有三大有利条件。一是拥有利好的政策机遇。中央扩大内需政策可使我市的基础设施和民生项目得到有力支持和加快建设，有利于我们扩大投资，进一步夯实发展基础；中央决定扩大国有林权制度改革试点，有利于我市乘势而上，进一步释放和拓展林改效应，减缓金融危机压力；我市被国家批准为首批资源枯竭城市之一，将连续4年享受国家财力性转移支付资金等政策支持，有利于我市经济转型产业和项目的加快发展；中央决定延长天保工程实施期限和建立生态补偿机制，省政府又将大小兴安岭生态功能区列入全省经济发展八大区域之一，并专门出台了实施意见，有利于我市加快建设林业生态体系和生态产业体系；国家实施适度宽松的信贷政策，有利于打开困扰我市多年企业发展壮大中的资金瓶颈。这些利好政策如能全部争取到手，用足、用好、用活，我市必将早日摆脱危机的困扰，步入加快发展的历史“拐点”，迎来加快发展的春天。二是拥有良好的发展基础和正确的发展思路。经过多年的实践探索，我市形成的以保护和恢复森林资源为基础，以林权改革为先导，以经济转型为路径，以振兴林区和致富人民为目标的生态经济社会可持续发展新模式，不仅使生态得到有效保护、经济得到较快发展、民生得到较大改善、社会保持和谐稳定，也与中央和省里的发展取向高度一致，与科学发展观的要求完全符合。只要我们坚定不移地走下去，我市的发展前景必将无限光明。三是拥有一笔宝贵的精神财富。林区开发60年来，我们伊春人战胜了无数的艰难险阻，创造了一个又一个奇迹。几代林区人薪火相传的知难而进、奋发向上的负重自强精神，勇争第一、敢为人先的开拓进取精神，同甘共苦、共克时艰的顾全大局精神，无时无刻不在激励我们。只要我们勇于在困难面前始终保持良好的精神状态，勇于把金融危机的紧逼加压转化为逆势而上的动力，善于在逆境中发现和培育有利因素，善于在矛盾相互转化中创造发展条件，咬住发展不放松，敢于在困难中大干苦干，就一定能够实现与全省、全国的同步甚至更快发展，创造出更加辉煌的业绩！

各位代表、同志们，今年市政府工作总体要求是：以邓小平理论和“三个代表”重要思想为指导，认真贯彻中央和省委省政府的重大部署及市委十届六次全会精神，全面落实科学发展观，深入实施“生态立市、产业兴市”战略，牢牢把握中央扩大内需等政策机遇，积极应对国际金融危机带来的风险和挑战，抓项目、保增长，抓林改、增活力，抓生态、强基础，抓联创、建名城，抓民生、促和谐，全力推进经济社会又好又快、更好更快发展。

今年全市经济社会发展的主要预期目标是：地区生产总值同比增长10%。地方财政收入按可比口径增长12%。规模以上工业增加值增长10%。全社会固定资产投资增长45%。社会消费品零售总额增长12%。国内引资与上年计划持平，实际利用外资与上年完成数持平，外贸进出口总额比上年增长15%。万元GDP综合能耗降低5%。城镇居民人均可支配收入增长8%，农民人均纯收入增长6%以上。城镇居民登记失业率控制在4.6%以内。

上述指标的确定，综合考虑了我市经济发展潜力和金融危机的影响，是审慎、留有余地的，但由于今年的不确定因素较多，经济下行趋势明显，要实现这些目标必须付出相当艰辛的努力。

（一）抓好生态功能区建设，全面提升生态建设水平。深入实施天保工程，认真落实生态功能区建设规划，促进生态建设的提档升级。继续加强防伐、防火、防垦工作。狠抓“严管林”，对破坏森林、破坏资源的行为始终保持高度警觉和高压态势，切实保护红松等珍贵树种，严防侵林毁湿开荒。继续扩大北部农林交错地区农民参与管护经营承包面积。实施“天眼工程”，在重点区域逐步推广应用卫星遥感和数字信息监控跟踪技术，使森林资源管护和防火的科学化水平再上一个大台阶。推广伊春区、朗乡、美溪等地水源热泵取暖、生物能源举炊等环保节能技术和生活方式，使烧柴改革有一个大突破。结合棚户区改造，加快推进林场所撤并，实施生态移民，建立生态无人区。超前做好停止木材主伐后的应对工作。积极配合省政府对上争取，力争在停伐补偿、木材加工企业原料林建设、金融债务减免等方面享受到更多政策。统筹安排、科学制定好过渡性措施，做好思想引导、人员转移、结构调整、产能整合等工作，确保在国家政策支持到位的情况下，全面停止木材主伐，做到主伐停下来、人员转出来、林子长起来。继续强化木材经销工作，确保在市场低迷的情况下，实现效益的最大化。切实加大森林资源培育力度。完善营林工作思路，重点加强森林经营和珍贵树种培育工作，促进红松天然更新，增加三大硬阔等优质材种育林比重，提高林分质量。认真落实省委、省政府“三年大造林”部署，大力推进农田防护、城镇、村屯、公路沿线等平原绿化工作。培育适合我市的绿化树种，努力使营林工作为我市乃至全省绿化作出更大贡献。抓好原料林基地建设，不断扩大红松果林、蓝莓浆果林等经济林基地建设，加快林果、林药、林板一体化进程，逐步提高林产工业原料供给能力。努力提升自然保护区建设水平。对现有的湿地自然保护区进行整合，改变孤岛化状态，提升保护功能，重点扶持五营、带岭等具备一定规模和水平的自然保护区晋升为国家级自然保护区。加快江河治理和退耕还林还湿步伐，使小兴安岭典型的生态系统和重要的野生动植物资源得到有效保护。积极推进地质灾害防治和矿山环境治理，建设更多的公园矿山和绿色矿山。

（二）抓好优势特色产业发展，不断加快经济转型步伐。全力抓好大项目建设。政策支持和项目建设是伊春发展与转型的两个基本点。今年，必须把大上项目和上大项目作为扩内需、保增长、调结构、促发展的主要手段，必须举全市之力争项目、上项目、管项目，必须把项目的争取和管理责任落实到位。重点抓好棚户区改造、林都机场、兴安湖水库、中心城区供水管网改造、中心城防洪工程、伊绥高速公路伊春段工程、电网改造、中心城污水处理厂和垃圾处理场、百湖工程等16个大项目。加强项目管理，做到每个项目、每个环节、每笔投资都经得起现实和历史的检验。做好新项目研究论证，丰富项目库，大幅度增加备选项目数量。加力发展接续替代产业。通过资源整合、要素重组、产业集聚，促进点状发展；通过建立基地，壮大龙头、做大品牌，促进集约发展；通过地域分工、个案规划、分类指导，促进增长极和发展带的隆起。从今年开始，将全力实施接续替代产业发展“4111工程”，力争经过三年左右的努力，建好“四大基地”，完善“一个体系”，打造“一区”、“一乡”。“四大基地”就是依托西林区，以西钢和金林矿业为龙头建设冶金基地；依托南岔区，以浩水、浩化为龙头建设建材化工基地；依托中心城区和铁力市，以升辉酒业、翔宇集团、新世纪食品、惠加贝乳业、葵花药业等企业为龙头建设森林食品、北药加工基地；依托中心城区、嘉荫口岸和双丰，以光明、双丰、友春等品牌为龙头，在整合林产工业资源的基础上，建设木材精深加工基地。“一个体系”就是着力构建市域内以嘉荫、伊春区、铁力、南岔为节点，辐射城乡、连接省内外、货畅其流的物流体系。“一区”、“ 一乡”就是把我市打造成为全国“森林旅游度假集合区”、全国的“绿色能源之乡”。冶金基地建设，围绕三年产钢能力达到600万吨目标，今年重点推进西钢180平方米烧结机、100万吨高速线材生产线和西林80万吨球团等项目建设进度，争取尽快竣工投产。同时，不断加大矿产资源勘察开发力度，提高原料保障能力。建材化工基地建设，围绕三年500万吨水泥产量目标，今年要全力抓好浩水日产5000吨生产线扩容改造。同时，促成浩化30万吨合成氨、52万吨尿素项目尽快上马。森林食品与北药基地建设，今年突出抓好食用菌产业素质提升行动和山野菜、山野果、乳制品、肉制品、农副产品及北药等的加工转化，叫响小兴安岭绿色、有机、原生态食品品牌。大力推行生态化、无毒化种植养殖模式，控制农药使用。加强对森林食品、北药产品的质量安全监管，建设大小兴安岭规模最大、功能最全的食品药品监测检疫中心，为森林食品、药品产业提供可靠的技术质量保证。木材精深加工基地建设，要推进家具、人造板、小木制品企业实施战略重组，开发科技含量高的终端产品，以质补量，以附加值补资源，开展林产工业“二次创业”。充分发挥光明家具的龙头作用，进一步扩大其市场份额，力争年内实现满负荷生产，带动全市木材精深加工行业做大做强。物流体系建设，今年要建成立足大小兴安岭、带动全省、辐射全国的森林产品批发大市场，以此推进跨区物流、跨国物流发展。森林旅游度假集合区建设，要抓住今年国家开展“生态旅游年”活动、我省北国风光特色旅游开发区、汤旺河国家公园建设的机遇和我市机场通航的契机，提升伊春的名气、人气、财气，推动全市的旅游业迈上一个大台阶。今年要在巩固夏季旅游的基础上，加大冬季旅游产品的开发力度，打响秋冬季旅游品牌。加强高档宾馆、酒店等基础设施配套建设，规范旅行社和景区经营秩序，提高服务质量和接待能力。采取延长商业网点营业时间等方式，使全市的商业文化活动与社会行为习惯尽快适应旅游城市发展的需要。全年要实现旅游收入19亿元，增长25.8%。绿色能源之乡建设，要积极引进战略投资者，扩大开发规模。今年积极推进金山屯大白山、老白山、带岭东山等风电和汤旺河干流梯级开发等水电项目建设，风电、水电装机容量预计各新增30.8万千瓦和1万千瓦，总量分别达到43.21万千瓦和1.424万千瓦。竭力扶持企业发展。深入开展“百日攻坚保增长”活动，加强对重点企业和项目经济运行的监控分析与协调，逐企业、逐项目、逐问题地研究制定帮扶措施，引导和帮助企业调整经营策略和产品结构，加快市场开拓、技术改造和产品创新步伐，全面提升应变能力和竞争能力，增强发展后劲。

（三）抓好改革开放，努力增强经济社会发展活力。加快推进国有林权制度改革向纵深发展。切实保护和经营好先期改革的8万公顷林地，加大扶持和服务力度，帮助承包户因地制宜地确定发展项目，搞活林地经营。支持林改户搞好病虫鼠害防治、森林防火和林间道路建设，巩固试点成果。逐步完善配套改革，探索解决好活立木交易市场启动等制约试点深入推进的关键性问题，为承包户长期经营好林地创造条件。认真做好宣传发动、调查设计、资产评估等各项扩大试点的准备工作，全力争取扩面，一旦国家批准，立即启动实施，力争第一个扩大。继续搞好国有企业改革。认真做好晨明水泥厂和带岭林业造纸厂政策性破产后的善后处理工作，指导浩水公司尽快改制到位。推进光明公司股改重组年内取得实质性进展。进一步深化行政管理体制改革。按上级要求，积极稳妥地推进政府机构改革，理顺部门关系，强化部门责任，着力解决职责交叉、权责脱节、推诿扯皮等问题。进一步探索分离企业办社会的途径和办法，加快社企分开步伐。根据全省安排，同步推进燃油税替代公路收费改革，妥善做好人员安置和贷款偿还工作。全方位推进对外开放。深入实施“南联北开”战略，根据我市发展需要确定招商重点领域和项目，创新招商方式，提高引资的针对性和投资的稳定性。强化区域合作，做好参会办节活动。加快推动对外经贸合作提档升级，积极开拓新兴市场，调整优化出口商品结构，扩大地产品出口比重。重点推进对俄深度开发合作，制定实施《伊春市沿边开发开放规划》，在伊春区建成全职的海关和检验检疫局，搞好嘉荫口岸基础设施建设，做好停止主伐后进口木材替代的政策准备工作，千方百计增加木材、铁矿石的进口和钢材、水泥等优势产品出口。

（四）抓好新农村建设，改善农村和林场所生产生活条件。切实加强耕地、基本农田保护和优质粮食生产。重点抓好标准化生产技术和专用玉米、大豆、水稻品种的普及推广，粮食作物优质品种普及率、标准化覆盖率达到90%以上。充分发挥铁力市省级农业科技示范园区的带动作用，大力开展粮食高产创建活动，努力提高单位面积产量。加快推进农业、森工多种经营产业化、品牌化。积极培育发展加工型和销售型产业龙头，加强生产基地建设，通过龙头带基地，基地带农户，促进农民增收致富。加快有机食品、绿色食品和无公害农产品的认证步伐，打造有机绿色食品品牌，促进现代农业发展和林场经济转型。深化场县合作。创新共建模式，重点抓好嘉荫县10万亩承包经营土地向国有农场的流转工作，铁力市与铁力农场万亩水稻示范区建设，实现场县从机械共用、技术共享向土地流转和利益一体化转变，提升共建水平。启动第二批新农村建设试点工作。以保障农产品有效供给、促进农民收入稳定增长为目标，加大对新农村试点的投入，认真组织开展新一轮帮建活动，重点办好“水、电、路、房、医、教、能、保”等公共事业，积极扶持农民创业就业。全年农业增加值同比增长6%。

（五）抓好基础设施和环境建设，进一步完善城市功能。科学搞好城市规划，引导城乡建设健康有序发展。从交通、供热、供水、垃圾污水处理等城市基础要素入手，加快“四区连片”建设步伐，扩大城市容量，集聚城市人气，不断提升城市品位。大力加强交通建设。按照省公路建设“三年决战”的要求，抓好伊绥高速伊春段、黑嘉公路嘉荫至黑河段、绥嘉公路嘉荫至绥滨段等公路建设，完成友好至翠宏山铁矿、红星至大平台等670公里的农村公路建设。高质量地完成林都机场建设，确保9月份通航。争取将汤嘉铁路列入省和铁道部的规划，早日开工建设。继续推进安居工程建设。结合林场所布局调整、旅游风光道建设和中心城扩容，按照宜平则平、宜楼则楼的原则，全力推进棚户区、农村泥草房改造，使棚改收到改善群众住房、促进生态建设、做大房地产市场、改变城乡面貌的多重效果。大力推进廉租房建设和实物配租力度，让困难群众、低保对象尽快住上安全卫生的廉价房。坚持节约、集约用地，积极培育房地产业，通过机场通航、棚改、垃圾污水处理设施建设、中心城防洪工程建设等，打造宜居城市，带动房地产市场升温。积极实施“三供两治”工程。努力保障群众对供水、供热、供气的需求和垃圾、污水无害化处理的需求，自来水普及率新增2个百分点，达到62%，集中供热普及率新增4个百分点，达到50%，创造条件，让更多的城乡人口用上液化气。大力推进华能热电厂的筹建，力争年内动工。加快中心城污水处理厂、垃圾处理场建设进度，确保今年8月投入使用。加快兴安湖水库建设，确保四季度试运行。切实加快电网改造。整合电力市场，改善供电环境，开工新建2个220千伏、3个110千伏变电所工程，加大老旧设备改造力度，确保电网安全。努力搞好小城镇建设。进一步完善小城镇住宅、排水、环卫等基础设施，增加家庭旅馆数量，改善质量，开展家庭宾馆挂星活动，打造特色旅游城镇。协调推进“四城联创”。重点综合整治市容环境卫生，加强传染病防治、食品药品安全监管和水质及大气环境的监测，继续加大城市的保洁力度，增加水冲式公厕的数量，提速创建全国卫生城市，力争今年达标挂牌。深入开展精神文明创建活动，着力提高市民文明素质，努力营造团结和谐、健康向上的人文环境，力争两年内建成全国文明城市。大力加强城市园林绿化美化和景点景观建设，增加城市亮点，提升城市形象，打造林城独特魅力，力争早日建成国家生态园林城市和森林城市。今年提高全市绿地绿化覆盖率0.3个百分点，达到7117.9公顷。加强对“四城联创”工作的宣传，教育和引导广大群众养成良好的行为习惯，自觉地支持参与创建工作。

（六）抓好财政金融工作，提高财政金融保障能力。加大保增长、保民生的财政支持力度。财政要重点向民生工程、“三农”、重大基础设施、生态建设、林权改革、结构调整、自主创新等重要领域倾斜投入，放大财政资金的带动效应。充分发挥财政资金的引导作用。做大政府担保基金，建立以贴息、担保、参股和风险投资为主的财政产业性资金投入机制。落实好国家适度宽松的信贷政策，扩大信贷投放额度，扩大保险在经济领域尤其是农业、林业领域的覆盖面，努力解决优势产业、林权改革及中小企业发展中的资金瓶颈问题。努力改善金融生态环境。突出抓好社会诚信建设，健全市（县）区两级担保体系和资源共享的信用体系，坚决依法打击逃废金融债务的不法行为，提高防范金融风险的能力。建立对地方支持力度大的金融部门奖励制度。积极创新融资方式。探索以产权、市场、资产、土地、活立木等资源置换资金，发行地方、企业债券、开展森林碳汇等方式，吸引和鼓励社会力量及民间资本投向基础设施建设和经济领域、社会事业，努力通过财政、信贷、企业和社会性资金的多元投入，推动全市经济社会加快发展。高度重视税收征管工作。支持税务机关落实国家各项税收扶持政策，依法履行筹集财政收入、调节经济和收入分配职能，严肃查处偷、逃税案件，切实维护税收政策的统一性和严肃性。对贡献突出的纳税大户给予奖励。

（七）抓好社会建设，竭力保障和改善民生。大力发展各项社会事业，尽心竭力地解决好人民群众最关心、最直接、最现实的利益问题。今年财政困难很大，但无论如何，都将必保增加对民生的投入，必保提高困难群众的生活水平，必保扩大民生工程的覆盖面，让公共财政的阳光普照全市人民。进一步抓好教育科技事业的发展。继续整合基础教育资源，不断改善办学条件，积极推进中小学校标准化信息化建设，强化教师培训，提高教育质量，巩固提高“双基”成果，促进幼儿教育、义务教育、高中阶段教育和高等教育均衡发展。大力发展职业教育，建设公共实训基地，尽可能为伊春的发展培养更多的实用型人才。大力实施“人才强市”战略，坚持引育并举，逐步满足经济社会发展对人才的需求。加大科研和科技成果推广力度，建立科技创新表彰奖励制度，对在生态建设、食用菌培育、生物和高新技术产业等我市重点发展领域做出突出科技贡献的集体和个人，给予重奖。继续改善公共卫生服务。积极推进医疗体制改革，进一步建立健全基本医疗卫生制度，建设覆盖城乡居民的公共卫生医疗服务体系，努力为群众提供安全、便利、廉价的公共医疗卫生服务。坚持综合性医院做大做强、专科医院体现特色的原则，逐步整合设备和人力资源，搞好各级各类医疗卫生机构建设，着力完善林场所、农村三级卫生服务网络和社区卫生服务中心建设。同时，认真落实基本国策，继续做好人口与计划生育工作，解决好林场所大龄职工的婚姻问题。积极发展文体事业。推进文化惠民工程，重点建好森林博物馆和群众艺术馆，加强文艺精品创作，搞好乡镇村屯、场所文化站建设。大力开展群众性文化活动，积极推进有线电视数字化建设，不断满足人民群众的精神需求。继续发展群众体育事业，加强体育健身场所建设，积极做好争办省十三届运动会工作。适应旅游业发展需要，创新体育项目，开展特色趣味性体育活动。扎实推进城乡基层政权建设。进一步加强城市社区建设，全面启动农村社区建设。继续优化社区建设布局，不断加大投入，力争三年内我市社区基础设施建设有较大改观。充分调动社区内各种力量参与社区建设，推进社区资源的共建共享。努力抓好社区服务者队伍和基层民主政治建设，进一步夯实政权基础。认真落实双拥和优抚政策，加强边防和国防后备力量建设，积极做好复转军人安置工作，不断加强对优抚对象的抚慰、救济。进一步做好妇女儿童、民族宗教、国家安全、防震减灾、人防等工作。千方百计扩大就业再就业。认真落实就业政策，全面推动创业带动就业，大力开发公益性岗位，帮助就业困难群体尽快实现就业再就业。充分发挥林改和职工自营经济在促进林业职工就业增收和林区社会稳定方面的作用，引导更多职工群众通过参与林改、发展自营经济实现就业再就业和脱贫致富。结合旅游业发展和企业用人需求，开展旅游业创业培训活动。积极稳定现有就业岗位，鼓励和支持困难企业采取在岗培训、轮班工作等办法，尽量不裁员或少裁员。不断提高社会保障能力。加快完善城乡社会保障体系，进一步扩大城镇职工基本养老保险、基本医疗保险和生育保险覆盖面，强化基金征缴和管理，建立健全城乡居民低保与物价变动相适应的动态调整机制。加强社会救灾救济、救助和法律援助工作，保障困难群众等弱势群体的基本生活权益。加强和改进价格调控，确保居民消费价格涨幅控制在全省平均水平以下。全力维护社会稳定。深入开展“平安伊春”建设，强化“五五”普法，完善社会治安综合治理工作机制，不断提升对各类违法犯罪活动的打击和防范能力，争取实现全国社会治安优秀地市 “二连冠”。加强安全生产工作，强化对非煤矿山、道路交通、消防、工程建筑、公共场所及易燃易爆等重点行业领域的专项治理和监管，强化责任落实和追究，坚决遏制重特大安全事故的发生。认真做好信访工作，深入开展矛盾纠纷排查和调处工作，以高度负责的精神对待人民群众的诉求，努力理顺情绪，化解矛盾。

今年将重点为人民群众办好10件实事。1、全年实现就业再就业4.5万人，新增就业3.7万人。为林改户提供贷款担保和贴息，通过发展林下经济，吸纳更多人就业。2、改造棚户区75万平方米，农村泥草房2200户，让4.1万户困难居民住上廉租房。3、为13个政企合一区和4个林业局在职职工增加工资，为公务员增加津贴补贴，提高职工收入水平。4、将历史拖欠的项目内养老金补发到位，不断提高养老保险水平。5、启动城镇基本医疗保险，实现对城镇居民的全覆盖。6、加强农村和林场所饮水安全工程建设，解决3万人饮水安全问题。7、深入开展第四轮扶贫解困活动，确保扶贫对象按时脱贫。8、改造中心城区6.2公里供热管网，提高城市供热质量。9、分批更新公交运营车辆，将线路延伸至翠峦区，改善中心城区的公共交通状况。10、建设10个文化、体育等项功能齐全的标准化中心社区。

（八）抓好政府自身建设，努力建设人民满意的政府。完成今年各项经济社会发展任务，对政府工作提出了新的更高的要求。市政府将始终如一地坚持科学施政、依法行政、从严治政、民主理政、廉洁从政的政府工作基本原则，深入推进学习型、创新型、实干型、亲民型、和谐型、廉洁型政府机关建设。努力提高行政效能。着力推动政府职能和管理向提供优质高效服务、创造良好发展环境、维护公平正义、强化社会管理和公共服务转变。严格按照法定权限和程序行使职权，不断加大政府法制监督力度，搞好行政复议工作，维护行政执法相对人的合法权益。全面推行政府绩效考评，对重点工作实行全程跟踪问效，强化行政问责和绩效管理，严肃查处政府工作人员决策执行不作为、乱作为和腐败行为，确保政府权力依法规范运行。深入推进服务机制创新，着力办好行政服务中心，重心前移，把更多的行政资源放到前台。有效落实审批事项纳入制度，精简优化审批项目和环节，严禁行政审批“体外循环”，推行限时并联审批和超时默许制，构建名副其实的“一站式”审批服务体系。积极推行政务公开，加快电子政务建设，办好政府门户网站，年内要推出一批网上办公项目。自觉接受内外监督。依法接受市人大及其常委会监督，积极支持人民政协履行职能，认真办好人大代表建议和政协委员提案，主动听取各民主党派、工商联和各人民团体的意见、建议，对重大工作决策和部署，利用各种传媒，广泛征求全市人民的意见建议，接受群众监督，不断提高各项工作决策的民主化、科学化水平。切实增强民本思想。进一步转变作风，大力弘扬艰苦奋斗精神，厉行勤俭节约，反对铺张浪费，大幅压缩会议讲话、政府文件、临时机构、公费出国出境和公务消费，确保行政办公经费“零增长”。办好市长信箱和市长热线，认真听取社情民意，及时了解群众的需求和呼声，及时宣传政策和决策、解疑答惑，及时帮民所需，解民所难，努力使政府提出的发展思路、做出的工作部署、出台的政策措施，更加符合伊春的实际，更加符合人民群众的愿望。

各位代表、同志们，展望本届政府的后三年工作，正是扩大内需政策的实施期，林权改革的扩大期，资源型城市转型的关键期，天保工程的延伸期，生态功能区建设的全面启动期。走好这一步，伊春将彻底摆脱“两危”的阴影，踏上全面协调可持续发展的伟大征程，祖国林都必将走向复兴，再创辉煌。让我们在市委的坚强领导下，紧紧依靠全市人民，振奋精神、坚定信心，抓住机遇、攻坚克难，同心同德、大干快上，努力夺取2009年全市经济社会发展的新胜利，以优异的成绩向新中国成立60周年献礼！
